# Layered Concept Lattice Model and Its Application to Build Rapidly Concept Lattice

**DOI:** 10.1155/2020/5784209

**Published:** 2020-06-11

**Authors:** Xia Wu, Jialu Zhang, Jiaming Zhong

**Affiliations:** ^1^College of Mathematics and Finance, Xiangnan University, Chenzhou 423000, China; ^2^College of Economic and Management, Xiangnan University, Chenzhou 423000, China

## Abstract

When some attributes of a formal context can be decomposed into some subattributes a model of layered concept lattice to improve the efficiency of building concept lattice with complex structure attribute data is studied, the relationship between concept lattice and layered concept is discussed. Two algorithms are proposed: one is the roll-up building algorithm in which the upper concepts are built by the lower concept and the other is the drill-down algorithm in which the lower concepts are built by the upper concept. The examples and experiments show that the layered concept lattice model can be used to model complex structure attribute data, and the roll-up building algorithm and the drill-down algorithm are effective. The layered concept lattice model expands the scope of the research and application of concept lattice, the roll-up building algorithm, and drill-down algorithm of layered concept lattice to improve the efficiency for building concept lattice.

## 1. Introduction

Humans usually describe and recognize objective things from different levels and different granularity. There is a process of deepening the attribute characteristics of objective things, and hence the knowledge concepts at different levels or at different granularity are obtained [1, 2]. Granular computing is a kind of useful mathematical method for processing complex structure data, and the idea of granular computing fits perfectly with the hierarchical and granular thinking mode of “from coarse to fine, from whole to part” in the process of human cognition [3–5]. The idea of granular computing originated from professor Zadeh [6]. Since Lin summarized relevant studies and introduced the term granular computing in 1998 [7], the thinking and methods of granular computing have appeared in many fields, such as rough set, fuzzy set, evidence theory, cluster analysis, machine learning, data mining, and knowledge discovery. In recent years, research studies on granular computing have been extensive and many meaningful results have been obtained. For example, Yao studied the basic problems and methods of granular computing [3], Lin studied the granular structure and representation [7], Pedrycz studied the granular computing methodology, mathematical framework, and information granulation algorithm [8], and Zhang and Zhang studied the quotient space [9].

Concept lattice is the key data structure of formal context analysis, and it is also a kind of basic data structure suitable for knowledge representation and knowledge discovery [10]. There have been many studies on basic theory of concept lattice [11] and algorithms to accelerate the construction of concept lattice [12–14], and concept lattice has been widely applied in many fields including data mining [15], knowledge discovery [16], information retrieval, and information extraction [17, 18]. Theory and application research of concept lattice are inseparable from the structure analysis of concept lattice and the model extension of concept lattice. The existing concept lattice models and their extension models are established on the relation between objects and attributes, they handle only the simple type of data, and there is no model that has been able to handle data with complex structures. For example, by using a fuzzy set on the basic language set [19], Wolff represented attribute values with fuzzy language variable values in a formal context, classified objects according to the scale, and then built concept lattice based on the scale [20]. Burusco and Fuentes discussed the concept lattice structure based on L-fuzzy concept set and proposed a method to build concept lattice in this case [21]. Considering that Burusco and Fuentes' models cannot handle continuous membership values and have high computational complexity, Qiang et al. proposed a fuzzy conceptual lattice model that can handle continuous membership values, and based on this model, they discussed the extraction of fuzzy rules and the clustering of fuzzy concepts [22]. Ali and Samir introduced the real interval formal context, established the real interval concept lattice, and built the classifier on the real interval concept lattice for data classification [23]. Qu et al. aimed to establish the relationship between formal concept analysis and rough set theory, obtained derivative formal context that can be induced by the notion of nominal scale and plain scaling and the technique of plain scaling in an information system, and proved that some core notions in rough theory such as partition, upper and lower approximation, independence, dependence, and reduction can be reinterpreted in derivative formal context. They pointed out that the rough set model can be extended by using plain scaling, but they have not further studied the concept lattice structure in the derivative formal context [24].

Due to the complexity of research objects, complex structural data are widely used in practical applications. For example, in the field of network intelligent information processing, it is necessary to analyze the correlation among characters, time, action, environment, and other elements through text, image, audio, video, etc. These elements are usually expressed in different types of data [25]. In particular, under big data environment, the remarkable feature of data is the diversity of types and the complexity of structures. An application often deals with both structured data and semistructured, unstructured data such as text, images, audio, video, video, and Web. In order to apply formal concept analysis to study knowledge representation and knowledge discovery in big data environment, the first thing to do is to extend the existing concept lattice model. In the process of extending the concept lattice model in big data environment, the attribute values used to describe the characteristics of unstructured data, such as text, images, audio, video, and Web, include word values, text values, vector values, and their composite values, in addition to the usual number value and character value [26, 27]. On formal context analysis on complex structural data, Zhi proposed a generalized concept lattice model for heterogeneous data analysis by discussing the partial order composition on heterogeneous datasets [28, 29]. However, Zhi's concept lattice model for heterogeneous data is only considered at the same level and at the same granularity, which does not reflect the idea of hierarchy and granularity of human thinking. Therefore, to describe human thinking process from a mathematical perspective or to apply the concept lattice theory to describe the human thinking process, we need to build the concept lattice model at different levels and different granularity.

In order to satisfy the need for describing in detail the knowledge concept on unstructured dataset, the thinking of granular computing is applied to the formal concept analysis of data with complex structure. The layered structure of attribute data is fully considered, and a layered concept lattice model with layered and three-dimensional structure is built in the complex structure data environment. The relation between concept lattice of original formal context and layered concept lattice of layered formal context is discussed. The roll-up building algorithm in which the upper concept is built by the lower concept and the drill-down algorithm in which the lower concept is built by the upper concept are proposed. The examples and experiments show that the roll-up and the drill-down algorithms are effective.

## 2. Preliminary

In this section, we summarize some basic notions and conclusions on concept lattices. For more details of these notions and conclusions, we refer the reader to Ganter and Wille's works “Formal Concept Analysis” [10].

**Definition 1 .**

A formal context is a triplet *K*=(*G*, *M*, *I*), where *I*⊆*G* × *M* is a binary relation between *G* and *M*. The elements in *G* and *M* are called objects and attributes, respectively. (*g*, *m*) ∈ *I* or *gIm* indicates the object *g* has the attribute *m*.

**Definition 2 .**

Let *K*=(*G*, *M*, *I*) be a formal context. For a set *A*⊆*G* of objects, we define a set of attributes common to all objects in *A* as

(1) $$\begin{array}{c} \alpha \left(A\right)=\left\{\left.m\in M\right|gIm,\;\forall g\in A\right\}. \end{array}$$

Correspondingly, for a set *B*⊆*M* of attributes, we define the set of objects that have all attributes in *B* as

(2) $$\begin{array}{c} \beta \left(B\right)=\left\{\left.g\in G\right|gIm,\;\forall m\in B\right\}. \end{array}$$

In brief, we also denote *α*({*a*}), *β*({*m*}) as *α*(*a*), *β*(*m*).

**Proposition 1 (see [10]).**

For *A*, *A*_1_, *A*_2_⊆*G* and *B*, *B*_1_, *B*_2_⊆*M*,

1. *A*_1_⊆*A*_2_⟹*α*(*A*_2_)⊆*α*(*A*_1_), *B*_1_⊆*B*_2_⟹*β*(*B*_2_)⊆*β*(*B*_1_)
2. *A*⊆*β*(*α*(*A*)), *B*⊆*α*(*β*(*B*))
3. *A*⊆*β*(*B*)⟺*B*⊆*α*(*A*)
4. *α*(*A*_1_ ∪ *A*_2_)=*α*(*A*_1_)∩*α*(*A*_2_), *β*(*B*_1_ ∪ *B*_2_)=*β*(*B*_1_)∩*β*(*B*_2_)
5. *α*(*A*_1_∩*A*_2_)⊇*α*(*A*_1_) ∪ *α*(*A*_2_), *β*(*B*_1_∩*B*_2_)⊇*β*(*B*_1_) ∪ *β*(*B*_2_)
6. (*β*(*α*(*A*)), *α*(*A*)) and (*β*(*B*), *α*(*β*(*B*))) are concepts

**Definition 3 .**

Let *K*=(*G*, *M*, *I*) be a formal context. For *A*⊆*G* and *B*⊆*M*, if *α*(*A*)=*B* and *β*(*B*)=*A*, then a pair (*A*, *B*) is called a formal concept of formal context *K*, and *A* and *B* are called the extent and the intent of (*A*, *B*), respectively. The set of all concepts of formal context *K* is denoted as *C*(*K*).

**Definition 4 .**

Let (*A*_1_, *B*_1_) and (*A*_2_, *B*_2_) be two formal concepts of a given formal context *K*. (*A*_1_, *B*_1_) is called a subconcept of (*A*_2_, *B*_2_) if *A*_1_⊆*A*_2_(or equivalently *B*_1_⊇*B*_2_), which can be denoted by (*A*_1_, *B*_1_) ≤ (*A*_2_, *B*_2_).

Obviously, the subconcept relation ≤ is a partial order on *C*(*K*). Since ≤ is a partial order, we can adopt the definition of neighboring nodes of order theory here. Let *C*_1_ and *C*_2_ be two concepts of a given formal context *K*. We say *C*_1_ is a lower neighbor (or a child) of *C*_2_ and *C*_2_ is an upper neighbor (or a parent) of *C*_1_, if *C*_1_ ≤ *C*_2_ and there is no other concept *C*_3_ with *C*_3_ ≠ *C*_1_, *C*_3_ ≠ *C*_2_, and *C*_1_ ≤ *C*_3_ ≤ *C*_2_.

**Theorem 1 (see [10]).**

The set *C*(*K*) of all formal concepts of context *K* together with partial order ≤ makes a complete lattice, which is called a concept lattice of *K* and is denoted as (*C*(*K*), ≤); the upper and lower bound operations are

(3) $$\begin{array}{c} \wedge_{t\in T}\left(A_{t},B_{t}\right)=\left(\cap_{t\in T}A_{t},\alpha \left(\beta \left(\cup_{t\in T}B_{t}\right)\right)\right),\\ \vee_{t\in T}\left(A_{t},B_{t}\right)=\left(\beta \left(\alpha \left(\cup_{t\in T}A_{t}\right)\right),\cap_{t\in T}B_{t}\right). \end{array}$$

## 3. Layered Concept Lattice Model and Its Roll-Up Algorithm to Build the Upper Concept Lattice

According to the analysis in the introduction, when we discuss the concept lattice theory and application of complex data structure, the problem of data attribute stratification is often encountered. For example, on the basis of the discussing new energy vehicles at coarse-grained, we often need to discuss the problem of new energy vehicles at a finer granularity to meet the needs of practical application: hybrid vehicles and pure electric vehicles. In terms of the concept lattice, when the new energy attribute is decomposed into lower hybrid power and pure electric power attributes, we need to analyze the formal concept in the layered formal context.

In this section, we give the definition of layered formal context and layered concept lattice, discuss their properties, and propose a roll-up building algorithm of layered concept lattice in a normal layered formal context.

### 3.1. Layered Concept Lattice Model and Roll-Up Algorithm Theory

**Definition 5 .**

Let *K*=(*G*, *M*, *I*) be a formal context. If every attribute in *M* can be represented as a subset, which means that

(4) $$\begin{array}{c} M=\left\{m_{1},m_{2},\cdots ,m_{l}\right\},\\ m_{k}=\left\{m_{k1},m_{k2},\cdots ,m_{ki_{k}}\right\},\;k=1,2,\cdots ,l, \end{array}$$

then *K*=(*G*, *M*, *I*) is called a layered formal context and *m*_*k*_ is called a layered attribute. In a layered formal context *K*=(*G*, *M*, *I*), the lower attributes corresponding to the layered attribute *m*_*k*_ are denoted as

(5) $$\begin{array}{c} \bar{m_{k}}=\left\{\left(m_{k},m_{k1}\right),\left(m_{k},m_{k1}\right),\cdots ,\left(m_{k},m_{ki_{k}}\right)\right\}. \end{array}$$

If *i*_*k*_=1, then $\bar{m_{k}}=m_{k}$, which is actually not a layered attribute.

By using the formal context *K*=(*G*, *M*, *I*), we can introduce a new layered formal context $\bar{K}=\left(G,N,J\right)$, where $N=\cup_{k=1}^{l}\bar{m_{k}}$, and the relation *J* between objects and attributes is defined by *gJ*(*m*_*k*_, *m*_*ki*_)⟺*gIm*_*k*_. For the convenience of expression, we also call *K* the upper formal context, $\bar{K}$ the lower formal context, *C*(*K*) the upper concept lattice, and $C\left(\bar{K}\right)$ the lower concept lattice accordingly.

A layered formal context *K*=(*G*, *M*, *I*) is normal if for every layered attribute *gIm*_*k*_ iff there is a unique iff there is a unique lower attribute *m*_*ki*_ such that *gJ*(*m*_*k*_, *m*_*ki*_).

In this paper, we only discuss the normal layered formal context. The following conclusion is obvious.

**Proposition 2 .**

Suppose that *K*=(*G*, *M*, *I*) is a layered formal context. Then,

(6) $$\begin{array}{c} \left\{\beta \left(m_{k},m_{k1}\right),\beta \left(m_{k},m_{k2}\right),\cdots ,\beta \left(m_{k},m_{ki_{k}}\right)\right\}, \end{array}$$

is a partition of *β*(*m*_*k*_).

**Example 1 .**

Table 1 shows a simplified layered formal context *K*=(*G*, *M*, *I*), where *a*=“off-road vehicle,” *b*= “midrange vehicle,” *c*= “new energy power vehicle” which is a layer attribute, *c*_1_= “hybrid power,” and *c*_2_= “pure electric power.”

The formal context $\bar{K}$ induced by *K* is shown in Table 2.

When a formal concept *K* contains layered attributes, the upper concept lattice *C*(*K*) is closely related to the lower concept lattice $C\left(\bar{K}\right)$. Hence, we can start with the lower concept lattice $C\left(\bar{K}\right)$ by rolling up the lower attributes $\bar{m_{k}}$ of some lower concepts to an upper attribute *m*_*k*_ and then building a upper concept; this is easier than building the concept lattice directly from the formal context *K*.

**Theorem 2 .**

Suppose that *K*=(*G*, *M*, *I*) is a layered formal context, $\bar{K}=\left(G,N,J\right)$ is the corresponding lower formal context of *K*, and *C*(*K*) and $C\left(\bar{K}\right)$ are upper concept lattice and lower concept lattice, respectively. If $\left\{\left(A_{1},\bar{B_{1}}\right),\cdots ,\left(A_{\alpha },\bar{B_{\alpha }}\right)\right\}\subseteq C\left(\bar{K}\right)$ contain the same layered attribute $\bar{m_{k}}$ and are the nearest subconcepts of a node concept, then

1. $\left(A_{1},\bar{B_{1}}\right),\cdots ,\left(A_{\alpha },\bar{B_{\alpha }}\right)$ can be rolled up as a new concept (*A*^(*k*)^, *B*^(*k*)^), where *A*^(*k*)^=*A*_1_ ∪ ⋯∪*A*_*α*_ and $B^{\left(k\right)}=\left(\left(\bar{B_{1}}-\bar{m_{k}}\right)\cap \cdots \cap \left(\bar{B_{\alpha }}-\bar{m_{k}}\right)\right)\cup \left\{m_{k}\right\}$.
2. The lower concepts $\left(A_{i},\bar{B_{i}}\right)\left(i=1,\cdots ,\alpha \right)$ are all updated into $\left(A_{i},\left(\bar{B_{i}}-\bar{m_{k}}\right)\cup \left\{m_{k}\right\}\right)$, respectively.

**Proof**

It is worth pointing out before proving that a concept $\left(A_{i},\bar{B_{i}}\right)$ in $C\left(\bar{K}\right)$ may contain both nonlayered attributes and lower attributes in the form of (*m*_*k*_, *m*_*kl*_). In the following, we prove the theorem by four steps.

1. Prove equation *α*(*A*^(*k*)^)={*m* ∈ *M*|*gI* *m*, ∀*g* ∈ *A*^(*k*)^}=*B*^(*k*)^.

Let *m*_*l*_ ∈ *α*(*A*^(*k*)^). Then, for each *g* ∈ *A*^(*k*)^=*A*_1_ ∪ ⋯∪*A*_*α*_, we have *gIm*_*l*_. If *m*_*l*_=*m*_*k*_, then obviously *m*_*l*_ ∈ *B*^(*k*)^. If *m*_*l*_ ≠ *m*_*k*_, then because $\left(A_{i},\bar{B_{i}}\right)\left(i=1,\cdots ,\alpha \right)$ are concepts and *gIm*_*l*_ for each *g* ∈ *A*_*i*_, we have that $m_{l}\in \bar{B_{i}}\left(i=1,\cdots ,\alpha \right)$. So, $m_{l}\in \left(\bar{B_{1}}-\bar{m_{k}}\right)\cap \cdots \cap \left(\bar{B_{i_{k}}}-\bar{m_{k}}\right)$. This means that *m*_*l*_ ∈ *B*^(*k*)^. Hence,

(7) $$\begin{array}{c} \alpha \left(A^{\left(k\right)}\right)=\left\{\left.m\in M\right|gIm,\forall g\in A^{\left(k\right)}\right\}\subseteq B^{\left(k\right)}. \end{array}$$

Suppose that *m*_*l*_ ∈ *B*^(*k*)^. If *m*_*l*_ ∈ {*m*_*k*_}, then *m*_*l*_=*m*_*k*_. Hence, *m*_*k*_ are the layered attributes of concepts $\left(A_{1},\bar{B_{1}}\right),\cdots ,\left(A_{i_{k}},\bar{B_{i_{k}}}\right)$, so $\alpha \left(A_{i}\right)=\left\{\left.m\in N\right|gJm,\forall g\in A_{i}\right\}=\bar{B_{i}},\left(i=1,\cdots ,\alpha \right)$. Thus, for each *g* ∈ *A*^(*k*)^=*A*_1_ ∪ ⋯∪*A*_*α*_, there exists *i*_0_ such that *g* ∈ *A*_*i*_0__, so *gJ*(*m*_*k*_, *m*_*ki*_0__), and *gIm*_*k*_. This shows that *gIm*_*k*_ for each *g* ∈ *A*^(*k*)^=*A*_1_ ∪ ⋯∪*A*_*α*_. This means that *m*_*k*_ ∈ *α*(*A*^(*k*)^). If $m_{l}\in \left(\bar{B_{1}}-\bar{m_{k}}\right)\cap \cdots \cap \left(\bar{B_{\alpha }}-\bar{m_{k}}\right)$, then *m*_*l*_ ≠ *m*_*k*_ is not a layered attribute and $m_{l}\in \bar{B_{i}},\left(i=1,\cdots ,\alpha \right)$. Since $\left(A_{i},\bar{B_{i}}\right),\left(i=1,\cdots ,\alpha \right)$ are concepts, we have *gIm*_*l*_ for each *g* ∈ *A*^(*k*)^=*A*_1_ ∪ ⋯∪*A*_*α*_. Hence, *m*_*l*_ ∈ *α*(*A*^(*k*)^). Thus, *B*^(*k*)^⊆*α*(*A*^(*k*)^)={*m* ∈ *M*|*gIm*, ∀*g* ∈ *A*^(*k*)^}.

(2) Prove equation *β*(*B*^(*k*)^)={*g* ∈ *G*|*gI* *m*, ∀*m* ∈ *B*^(*k*)^}=*A*^(*k*)^.

At first, we prove *β*(*B*^(*k*)^)⊆*A*^(*k*)^. Let us assume *g* ∈ *β*(*B*^(*k*)^); then, for every $m\in B^{\left(k\right)}=\left(\left(\bar{B_{1}}-\bar{m_{k}}\right)\cap \cdots \cap \left(\bar{B_{\alpha }}-\bar{m_{k}}\right)\right)\cup \left\{m_{k}\right\}$, we have *gIm*. Hence by *gIm*_*k*_, we know that there are some *i* such that *gJ*(*m*_*k*_, *m*_*ki*_), so $gJ\bar{m_{k}}$, and let *i*_0_ be the subscript of biggest concept $\left(A_{i_{0}},\bar{B_{i_{0}}}\right)$ in these concepts, where the biggest concept means that the concept contains as many object elements as possible and as fewer attribute elements as possible. Thus, from $\left(A_{i_{0}},\bar{B_{i_{0}}}\right)$ being the biggest concept, we obtain that

(8) $$\begin{array}{c} g\in \beta \left(\bar{B_{i_{0}}}\right)=\left\{\left.g\in G\right|gJ\left(m_{k},m_{ki}\right),\forall \left(m_{k},m_{ki}\right)\in \bar{B_{i_{0}}}\right\}=A_{i_{0}}. \end{array}$$

This means that *g* ∈ *A*^(*k*)^. Therefore,

(9) $$\begin{array}{c} \beta \left(B^{\left(k\right)}\right)=\left\{\left.g\in G\right|gIm,\forall m\in B^{\left(k\right)}\right\}\subseteq A^{\left(k\right)}. \end{array}$$

Secondly, we prove *A*^(*k*)^⊆*β*(*B*^(*k*)^). Let *g* ∈ *A*^(*k*)^; then, there are some *i* such that *g* ∈ *A*_*i*_, and let *i*_0_ be the subscript of biggest concept $\left(A_{i_{0}},\bar{B_{i_{0}}}\right)$ in these concepts. Hence, $g\in A_{i_{0}}=\beta \left(\bar{B_{i_{0}}}\right)$, and for each $m\in \bar{B_{i_{0}}}$, *gJm*. This shows that *g* ∈ {*g* ∈ *G*|*gIm*, ∀*m* ∈ *B*^(*k*)^}, and then *g* ∈ *β*(*B*^(*k*)^). Therefore,

(10) $$\begin{array}{c} A^{\left(k\right)}\subseteq \beta \left(B^{\left(k\right)}\right)=\left\{\left.g\in G\right|gIm,\forall m\in B^{\left(k\right)}\right\}. \end{array}$$

From (1) and (2) we know that (*A*^(*k*)^, *B*^(*k*)^) is a concept.

(3) Prove that the concepts $\left(A_{i},\bar{B_{i}}\right)\in C\left(\bar{K}\right)\left(i=1,\cdots ,\alpha \right)$, which are not rolled up, are updated new concepts $\left(A_{i},\left(\bar{B_{i}}-\bar{m_{k}}\right)\cup \left\{m_{k}\right\}\right)\left(i=1,\cdots ,\alpha \right)$.

At first, we prove equation $\alpha \left(A_{i}\right)=\left\{\left.m\in M\right|gI\;m,\forall g\in A_{i}\right\}=\left(\bar{B_{i}}-\bar{m_{k}}\right)\cup \left\{m_{k}\right\}$.

Let *m*_*l*_ ∈ *α*(*A*_*i*_); then, for every *g* ∈ *A*_*i*_, we have *gIm*_*l*_. If *m*_*l*_=*m*_*k*_, then certainly $m_{l}\in \left(\bar{B_{i}}-\bar{m_{k}}\right)\cup \left\{m_{k}\right\}$. If *m*_*l*_ ≠ *m*_*k*_, then from that $\left(A_{i},\bar{B_{i}}\right)$ is a concept and *gIm*_*q*_ for every *g* ∈ *A*_*i*_, we have $m_{l}\in \bar{B_{i}}$. Hence, $m_{l}\in \bar{B_{i}}-\bar{m_{k}}$. This shows that $\alpha \left(A_{i}\right)=\left\{\left.m\in M\right|gIm,\forall g\in A_{i}\right\}\subseteq \left(\bar{B_{i}}-\bar{m_{k}}\right)\cup \left\{m_{k}\right\}$.

Conversely, let $m_{l}\in \left(\bar{B_{i}}-\bar{m_{k}}\right)\cup \left\{m_{k}\right\}$. If *m*_*l*_ ∈ {*m*_*k*_}, namely, *m*_*l*_=*m*_*k*_ is a layered attribute, then by $\left(A_{i},\bar{B_{i}}\right)$ being a concept, we obtain that

(11) $$\begin{array}{c} \bar{B_{i}}=\alpha \left(A_{i}\right)=\left\{\left(m_{k},m_{ki}\right)\in \left.N\right|gJ\left(m_{k},m_{ki}\right),\forall g\in A_{i}\right\}. \end{array}$$

Hence, by *gJ*(*m*_*k*_, *m*_*ki*_)⟺*gIm*_*k*_, we know *m*_*q*_ ∈ *α*(*A*_*i*_). If $m_{q}\in \bar{B_{i}}-\bar{m_{k}}$, then *m*_*q*_ ≠ *m*_*k*_ and $m_{q}\in \bar{B_{i}}$. Since $\left(A_{i},\bar{B_{i}}\right)$ is a concept, we have *gIm*_*q*_ for every *g* ∈ *A*_*i*_; hence, *m*_*q*_ ∈ *α*(*A*_*i*_). Therefore,

(12) $$\begin{array}{c} \alpha \left(A_{i}\right)=\left\{\left.m\in M\right|gIm,\forall g\in A_{i}\right\}⊇\left(\bar{B_{i}}-\bar{m_{k}}\right)\cup \left\{m_{k}\right\}. \end{array}$$

Secondly, we prove the following equation:

(13) $$\begin{array}{c} \beta \left(\left(\bar{B_{i}}-\bar{m_{k}}\right)\cup \left\{m_{k}\right\}\right)=\left\{\left.g\in G\right|gIm,\forall m\in \left(\bar{B_{i}}-\bar{m_{k}}\right)\cup \left\{m_{k}\right\}\right\}=A_{i}. \end{array}$$

Let $g\in \beta \left(\left(\bar{B_{i}}-\bar{m_{k}}\right)\cup \left\{m_{k}\right\}\right)$; then, *gIm* for every $m\in \left(\bar{B_{i}}-\bar{m_{k}}\right)\cup \left\{m_{k}\right\}$. Since $\left(A_{i},\bar{B_{i}}\right)$ is a concept, we know $\beta \left(\bar{B_{i}}\right)=\left\{\left.g\in G\right|gJm,\forall m\in \bar{B_{i}}\right\}=A_{i}$. Hence, by *gJ*(*m*_*k*_, *m*_*ki*_)⟺*gIm*_*k*_, we have

(14) $$\begin{array}{c} \beta \left(\left(\bar{B_{i}}-\bar{m_{k}}\right)\cup \left\{m_{k}\right\}\right)=\left\{\left.g\in G\right|gIm,\forall m\in \left(\bar{B_{i}}-\bar{m_{k}}\right)\cup \left\{m_{k}\right\}\right\}\subseteq A_{i}. \end{array}$$

Conversely, let *g* ∈ *A*_*i*_. Then, since $\left(A_{i},\bar{B_{i}}\right)$ is a concept, we have $\beta \left(\bar{B_{i}}\right)=\left\{\left.g\in G\right|gJm,\forall m\in \bar{B_{i}}\right\}=A_{i}$. If $m\in \bar{B_{i}}$ is not a layered attribute, then the relation between *g* and *m* does not change before and after rolling up. If $m\in \bar{B_{i}}$ is a layered attribute and its form is (*m*_*k*_, *m*_*kj*_), then by *gJ*(*m*_*k*_, *m*_*ki*_)⟺*gIm*_*k*_, we have *gIm* for each $m\in \left(\bar{B_{i}}-\bar{m_{k}}\right)\cup \left\{m_{k}\right\}$. Therefore,

(15) $$\begin{array}{c} A_{i}\subseteq \beta \left(\left(\bar{B_{i}}-\bar{m_{k}}\right)\cup \left\{m_{k}\right\}\right)=\left\{\left.g\in G\right|gIm,\forall m\in \left(\bar{B_{i}}-\bar{m_{k}}\right)\cup \left\{m_{k}\right\}\right\}. \end{array}$$

(4) Prove that the partial order among $\left(A_{i},\left(\bar{B_{i}}-\bar{m_{k}}\right)\cup \left\{m_{k}\right\}\right)\left(i=1,\cdots ,\alpha \right)$ is the same $\left(A_{i},\bar{B_{i}}\right)\left(i=1,\cdots ,\alpha \right)$.

By the structure of (*A*^(*k*)^, *B*^(*k*)^), for *i*=1, ⋯, *α*, we have

(16) $$\begin{array}{c} \left(A_{i},\left(\bar{B_{i}}-\bar{m_{k}}\right)\cup \left\{m_{k}\right\}\right)\leq \left(A^{\left(k\right)},B^{\left(k\right)}\right). \end{array}$$

If two concepts $\left(A_{i},\bar{B_{i}}\right)$ and $\left(A_{j},\bar{B_{j}}\right)$ are in $C\left(\bar{K}\right)$ with order $\left(A_{i},\bar{B_{i}}\right)\leq \left(A_{j},\bar{B_{j}}\right)$, then the following also holds:

(17) $$\begin{array}{c} \left(A_{i},\left(\bar{B_{i}}-\bar{m_{k}}\right)\cup \left\{m_{k}\right\}\right)\leq \left(A_{j},\left(\bar{B_{j}}-\bar{m_{k}}\right)\cup \left\{m_{k}\right\}\right). \end{array}$$

### 3.2. The Description and Analysis of Roll-Up Algorithm

In order to simplify the presentation below, we introduce the following two terms: parallel lower subconcepts and linear lower subconcepts. Let $\left(A_{1},\bar{B_{1}}\right),\cdots ,\left(A_{\alpha },\bar{B_{\alpha }}\right)$ be the lower subconcepts under node concept (*A*, *B*). If there is no partial order relation between each other, then $\left(A_{1},\bar{B_{1}}\right),\cdots ,\left(A_{\alpha },\bar{B_{\alpha }}\right)$ is called parallel lower subconcepts. If there is linear partial order relation among them, then $\left(A_{1},\bar{B_{1}}\right),\left(A_{1},\bar{B_{1}}\right),\cdots ,\left(A_{\alpha },\bar{B_{\alpha }}\right)$ is called linear lower subconcepts.

Because this paper only discusses the normal layered formal context and in a normal layered formal context an object will not have two lower attributes of one layer attribute, a concept will not contain more than two lower attributes except the empty concept (object set is empty set). Hence, every concept in the linear lower subconcepts only contains the same lower attribute, and different attributes among different subconcepts are nonlayer attributes. On the basis of this analysis, we can present a roll-up building algorithm of building a concept lattice based on attribute fusion. The process of rolling up from $C\left(\bar{K}\right)$ to *C*(*K*) involves the fusion of attributes, and the partial order in the concept lattice is defined according to the negative inclusion of the attribute subset. Thus, the rolling up is carried out from top to bottom, that is, start with the concept of having fewer attributes. The method of rolling up is to roll up the lower concept of node concept (*A*, *B*) in the lower concept lattice $C\left(\bar{K}\right)$ in a linear or parallel lower subconcepts, insert the new concept obtained from rolling up into the bottom of node (*A*, *B*), and update the node concept (*A*, *B*) and its related node concept accordingly (given in Algorithm 1).

The time complexity of Algorithm 1 is related to the number of layered attributes and the number of lower attributes in the layered context. In the following, we assume that the number of layered attributes in *K* is *l*, the number of node in $C\left(\bar{K}\right)$ is *s*, and the maximum number of lower attributes of all layered attributes is *r*. The computing number at Step 1 is *r*, and its time complexity is *O*(*r*). The time complexity at Step 2 is *O*(*r* log_2_ *r*); it is equal to the time complexity of *r* concept sorting. At Step 3, in the worst case, the number of linear lower subconcepts under a node in $C_{k}\left(\bar{K}\right)$ is *l*, and the number of computing in rolling up is *l*, but the total number of computing of all nodes in rolling up is not more than *s*. Hence, the time complexity at Step 3 is *O*(*s*). The time complexity of Step 4 and Step 5 is the same as that of Step 3, which is also *O*(*s*). The computing number at Step 6 does not exceed *s* − *r*, so the time complexity at Step 6 is *O*(*s* − *r*). The time complexity analysis at Step 7 has been included from Step 3 to Step 6. Therefore, the time complexity of Algorithm 1 is *O*((*r*+*r* log_2_ *r*+*s*+(*s* − *r*))*l*)=*O*(*l*(2*s*+*r* log_2_ *r*)).

The following example illustrates the application of the roll-up building algorithm in building concept lattice.

**Example 2 .**

**Figure 1:**
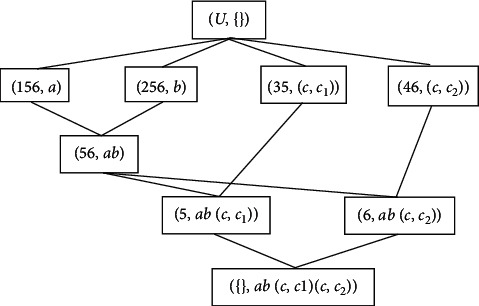
Concept lattice $C\left(\bar{K}\right)$ of formal context $\bar{K}$.

Consider the layered formal context *K* as shown in example 1; the induced formal context by *K* is shown in Figure 1.

**Step 1 s 1 and 2.**

The concepts containing lower attributes in $C\left(\bar{K}\right)$ are arranged in ascending order according to the number of attributes of concepts, $C_{k}\left(\bar{K}\right)=\left\{\left(35,\left(c,c_{1}\right)\right),\left(46,\left(c,c_{2}\right)\right),\left(5,ab\left(c,c_{1}\right)\right),\left(6,ab\left(c,c_{2}\right)\right),\left(\left\{\right\},ab\left(c,c_{1}\right)\left(c,c_{2}\right)\right)\right\}$.

**Step 2 .**

There are no linear lower subconcepts in this example.

**Step 3 .**

The concept of parallel lower concepts (35, (*c*, *c*_1_)), (46, (*c*, *c*_2_)) below node concept (*U*, {}) is rolled up into a new concept (3456, *c*) and inserted below node (*U*, {}). The upper bound node of the new node concept (3456, *c*) and the node concepts (156, *a*), (256, *b*) which do not contain lower attributes are also (*U*, {}).

The parallel lower concept (5, *ab*(*c*, *c*_1_)), (6, *ab*(*c*, *c*_2_)) below node concept (56, *ab*) is rolled up into a new concept (56, *abc*).

**Step 4 .**

After Step 4 is completed, there is no lower concept to be rolled up. There is only one layered attribute in this example, and the algorithm ends.

**Figure 2:**
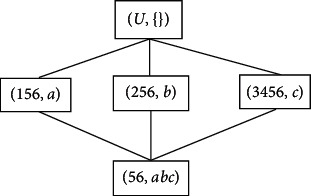
Concept lattice *C*(*K*) after rolling up.

Figure 2 shows the concept lattice *C*(*K*) obtained after the rolling up.

## 4. Drill-Down Algorithm to Build the Lower Concept Lattice

In this section, a drill-down algorithm to build lower layered concept lattice $C\left(\bar{K}\right)$ based on the upper concept lattice *C*(*K*) is presented.

### 4.1. Drill-Down Algorithm Theory

**Definition 6 .**

Let *K*=(*G*, *M*, *I*) be a layered formal context and *b* ∈ *M*. Then, (*β*(*b*), *α*(*β*(*b*))) is called an attribute concept.

**Proposition 3 .**

(*β*(*b*), *α*(*β*(*b*))) is the largest concept containing attribute *b*.

**Proof**

Obviously, (*β*(*b*), *α*(*β*(*b*))) is a concept containing attribute *b*. If (*A*, *B*) is another concept containing attribute *b*, then by {*b*}⊆*B* and Proposition 1, we have *β*(*b*)⊇*β*(*B*)=*A*. Hence, (*A*, *B*) ≤ (*β*(*b*), *α*(*β*(*b*))).

**Theorem 3 .**

Let *K*=(*G*, *M*, *I*) be a layered formal context and $\bar{K}=\left(G,N,J\right)$ be the lower formal context. Suppose that *m*_*k*_ is a layered attribute; then,

(18) $$\begin{array}{c} \bar{m_{k}}=\left\{\left(m_{k},m_{k1}\right),\left(m_{k},m_{k2}\right),\cdots ,\left(m_{k},m_{ki_{k}}\right)\right\}, \end{array}$$

is the lower attribute corresponding to *m*_*k*_, and (*β*(*m*_*k*_, *m*_*kj*_), *α*(*β*(*m*_*k*_, *m*_*kj*_))) are attribute concepts corresponding to the lower attributes (*m*_*k*_, *m*_*kj*_)(*j*=1,2, ⋯, *i*_*k*_). (*A*, *B*) ∈ *C*(*K*) is a concept and for every *j* ∈ {1,2, ⋯, *i*_*k*_}, there is no child (*X*, *Y*) of (*A*, *B*) with *A*∩*β*(*m*_*k*_, *m*_*kj*_)⊆*X*; then, for every *A*∩*β*(*m*_*k*_, *m*_*kj*_), *j* ∈ {1,2, ⋯, *i*_*k*_},

1. If *A*∩*β*(*m*_*k*_, *m*_*kj*_)=*A*, then (*A*, *B*) is updated to (*A*, (*B* − {*m*_*k*_}) ∪ {(*m*_*k*_, *m*_*kj*_)})
2. If *A*∩*β*(*m*_*k*_, *m*_*kj*_) ≠ *A*, then a new concept (*A*∩*β*(*m*_*k*_, *m*_*kj*_), (*B* − {*m*_*k*_}) ∪ {(*m*_*k*_, *m*_*kj*_)}) is added as a child of (*A*, *B*)

**Proof**

(1) is a special case of (2), so we only prove (2).

Firstly, we prove that if *A*∩*β*(*m*_*k*_, *m*_*kj*_) ≠ *φ*, then (*A*∩*β*(*m*_*k*_, *m*_*kj*_), (*B* − {*m*_*k*_}) ∪ {(*m*_*k*_, *m*_*kj*_)}) are concepts (*j*=1,2, ⋯, *i*_*k*_); hence, we need to prove two equations:

(19) $$\begin{array}{c} \alpha \left(A\cap \beta \left(m_{k},m_{kj}\right)\right)=\left\{\left.m\in N\right|gJm,\forall g\in A\cap \beta \left(m_{k},m_{kj}\right)\right\}\\ =\left(B-\left\{m_{k}\right\}\right)\cup \left.\left\{\left(m_{k},m_{kj}\right)\right\}\right), \end{array}$$

(20) $$\begin{array}{c} \beta \left(\left(B-\left\{m_{k}\right\}\right)\cup \left\{\left(m_{k},m_{kj}\right)\right\}\right)=\left\{\left.g\in G\right|gJm,\forall m\in \left(B-\left\{m_{k}\right\}\right)\cup \left\{\left(m_{k},m_{kj}\right)\right\}\right\}\\ =A\cap \beta \left(m_{k},m_{kj}\right). \end{array}$$

Since (*A*, *B*) is a concept, we know *α*(*A*)=*B*. By Proposition 1, we have that

(21) $$\begin{array}{c} \alpha \left(A\cap \beta \left(m_{k},m_{kj}\right)\right)⊇\alpha \left(A\right)\cup \alpha \left(\beta \left(m_{k},m_{kj}\right)\right)=B\cup \alpha \left(\beta \left(m_{k},m_{kj}\right)\right)⊇\left(B-\left\{m_{k}\right\}\right)\cup \alpha \left(\beta \left(m_{k},m_{kj}\right)\right). \end{array}$$

Conversely, let *m*_*q*_ ∈ *α*(*A*∩*β*(*m*_*k*_, *m*_*kj*_)). Then, for each *g* ∈ *A*∩*β*(*m*_*k*_, *m*_*kj*_), we have *gJm*_*q*_. In the following, we discuss it in two cases:

- $m_{q}\in \bar{m_{k}}$: if *m*_*q*_ ≠ (*m*_*k*_, *m*_*kj*_), then there is *i* ≠ *j* such that *m*_*q*_=(*m*_*k*_, *m*_*ki*_). By Proposition 2*β*(*m*_*k*_, *m*_*k*1_), ⋯, *β*(*m*_*k*_, *m*_*ki*_*k*__)} is a partition of *β*(*m*_*k*_), we know that for every *g* ∈ *β*(*m*_*k*_, *m*_*kj*_), *g* and (*m*_*k*_, *m*_*ki*_) have no relation *J*. This contradicts *gJm*_*q*_ for each *g* ∈ *A*∩*β*(*m*_*k*_, *m*_*kj*_). Thus, *m*_*q*_ ∈ {(*m*_*k*_, *m*_*kj*_)}.
- $m_{q}\notin \bar{m_{k}}$: if *m*_*q*_ ∉ *B* − {*m*_*k*_}, then obviously *m*_*q*_ ≠ *m*_*k*_, and hence *m*_*q*_ ∉ *B*. Because (*A*, *B*) is a concept containing attribute *m*_*k*_, there exists *g* ∈ *A* such that *g* and *m*_*q*_ have no relation *J*. The set of all elements of *A* that has no relation *J* with *m*_*q*_ is denoted as *A*_0_. If *A*_0_∩*β*(*m*_*k*_, *m*_*kj*_) is an empty set, then for any *g* ∈ *β*(*m*_*k*_, *m*_*kj*_) and *g* ∈ *A*, *g* and *m*_*q*_ have no relation *J*. This contradicts *gJm*_*q*_ for each *g* ∈ *A*∩*β*(*m*_*k*_, *m*_*kj*_). Hence, there is *g*_0_ ∈ *A*_0_∩*β*(*m*_*k*_, *m*_*kj*_) such that *g*_0_ and *m*_*q*_ have no relation *J*. This also contradicts *gJm*_*q*_ for each *g* ∈ *A*∩*β*(*m*_*k*_, *m*_*kj*_). Therefore, if $m_{q}\notin \bar{m_{k}}$, then *m*_*q*_ ∈ *B* − {*m*_*k*_}.

This proves that

(22) $$\begin{array}{c} \alpha \left(A\cap \beta \left(m_{k},m_{kj}\right)\right)=\left(B-\left\{m_{k}\right\}\right)\cup \left\{\left(m_{k},m_{kj}\right)\right\}. \end{array}$$

Since (*A*, *B*) is a concept, we have *β*(*B*)=*A*. By Proposition 1 we obtain

(23) $$\begin{array}{c} \beta \left(\left(B-\left\{m_{k}\right\}\right)\cup \left\{\left(m_{k},m_{kj}\right)\right\}\right)=\beta \left(B-\left\{m_{k}\right\}\right)\cap \beta \left(m_{k},m_{kj}\right)⊇\beta \left(B\right)\cap \beta \left(\left(m_{k},m_{kj}\right)\right)\\ =A\cap \beta \left(m_{k},m_{kj}\right). \end{array}$$

Conversely, let

(24) $$\begin{array}{c} g\in \beta \left(\left(B-\left\{m_{k}\right\}\right)\cup \left\{\left(m_{k},m_{kj}\right)\right\}\right)=\beta \left(B-\left\{m_{k}\right\}\right)\cap \beta \left(m_{k},m_{kj}\right). \end{array}$$

From *g* ∈ *β*(*m*_*k*_, *m*_*kj*_), we know *gJ*(*m*_*k*_, *m*_*kj*_). By *gIm*_*k*_⟺*gJ*(*m*_*k*_, *m*_*kj*_), we have *gIm*_*k*_. From *g* ∈ *β*(*B* − {*m*_*k*_}), we know *gIm* for each *m* ∈ *B* − {*m*_*k*_}. Hence, *gIm* for every *m* ∈ *B*. This shows that *g* ∈ *β*(*B*)=*A*, and hence *g* ∈ *A*∩*β*(*m*_*k*_, *m*_*kj*_). Therefore,

(25) $$\begin{array}{c} \beta \left(\left(B-\left\{m_{k}\right\}\right)\cup \left\{\left(m_{k},m_{kj}\right)\right\}\right)\subseteq A\cap \beta \left(m_{k},m_{kj}\right). \end{array}$$

This proves that

(26) $$\begin{array}{c} \beta \left(\left(B-\left\{m_{k}\right\}\right)\cup \left\{\left(m_{k},m_{kj}\right)\right\}\right)=A\cap \beta \left(m_{k},m_{kj}\right). \end{array}$$

Next, we prove that the partial order among concepts $\left(A_{i},\bar{B_{i}}\right)\left(i=1,\cdots ,\alpha \right)$ is the same as the partial order among $\left(A_{i},\left(\bar{B_{i}}-\bar{m_{k}}\right)\cup \left\{m_{k}\right\}\right)\left(i=1,\cdots ,\alpha \right)$.

At first, for *i*=1, ⋯, *α*, by the construction of (*A*^(*k*)^, *B*^(*k*)^), we have

(27) $$\begin{array}{c} \left(A_{i},\left(\bar{B_{i}}-\bar{m_{k}}\right)\cup \left\{m_{k}\right\}\right)\leq \left(A^{\left(k\right)},B^{\left(k\right)}\right). \end{array}$$

Secondly, if $\left(A_{i},\bar{B_{i}}\right)\leq \left(A_{j},\bar{B_{j}}\right)$ in $C\left(\bar{K}\right)$, then we also have

(28) $$\begin{array}{c} \left(A_{i},\left(\bar{B_{i}}-\bar{m_{k}}\right)\cup \left\{m_{k}\right\}\right)\leq \left(A_{j},\left(\bar{B_{j}}-\bar{m_{k}}\right)\cup \left\{m_{k}\right\}\right). \end{array}$$

### 4.2. The Description and Analysis of Drill-Down Algorithm

According to Theorem 3, started with the upper concept lattice *C*(*K*), inserted some new concepts, modified some concepts and deleted some concepts, we can obtained lower concept lattice $C\left(\bar{K}\right)$. In the following, we propose a drill-down algorithm to build lower concept lattice based on attribute decomposition (given in Algorithm 2).

The time complexity of Algorithm 2 is analyzed below. In the following, we assume that the number of layered attributes in *K* is *l*, the number of node concepts in *C*(*K*) is *s*, and the maximum number of lower attributes is *r*. The Bordat algorithm building concept lattice of formal context (*G*, *M*, *I*) is improved by Chen in [12], and the time complexity of the improved algorithm is less than *O*(|*G*||*M*|^2^). Then, in the worst case, the time complexity of Step 1 is *O*(|*G*|*r*^2^). The time complexity of Step 2 is *O*(*sr*). The time complexity of Step 3 is *O*(*r*^2^). Therefore, the time complexity of algorithm 2 is *O*((|*G*|*r*^2^+*sr*+*r*^2^)*l*)=*O*(*l*((|*G*|+1)*r*^2^+*sr*)).

The following example illustrates the application of the drill-down building algorithm in building the concept lattice.

**Example 3 .**

**Figure 3:**
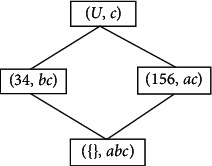
Concept lattice *C*(*K*) of context *K*.

A layered formal context *K*=(*G*, *M*, *I*) is shown in Table 3, and the concept lattice of *K* is shown in Figure 3. The lower formal context $\bar{K}=\left(G,N,J\right)$ induced by *K* is shown in Table 4.

**Figure 4:**
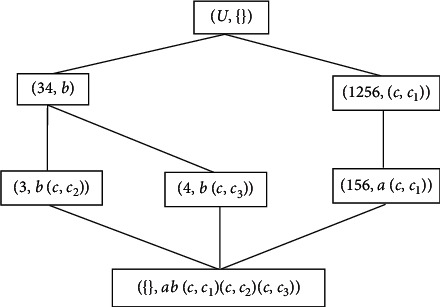
Concept lattice $C\left(\bar{K}\right)$ of formal context $\bar{K}$.

The concept lattice of $\bar{K}$ is shown in Figure 4.

**Step 5 .**

Layered attribute *c* has three lower attributes, and the corresponding lower attribute concepts are (1256, (*c*, *c*_1_)), (3, *b*(*c*, *c*_2_)), (4, *b*(*c*, *c*_3_)).

**Step 6 .**

There are four concepts in *C*(*K*) that contain the layered attribute *c*. According to Step 2 in Algorithm 2, (*U*, *c*) is updated into three new concepts (1256, (*c*, *c*_1_)), (3, *b*(*c*, *c*_2_)), (4, *b*(*c*, *c*_3_)); (34, *bc*) is updated into two new concepts (3, *b*(*c*, *c*_2_)), (4, *b*(*c*, *c*_3_)); (156, *ac*) is updated into a new concept (156, *a*(*c*, *c*_1_)).

**Step 7 .**

Two lower attribute concepts (3, *b*(*c*, *c*_2_)), (4, *b*(*c*, *c*_3_)) can be combined into a new concept (34, *b*) and inserted above (3, *b*(*c*, *c*_2_)), (4, *b*(*c*, *c*_3_)).

**Step 8 .**

There is only one layered attribute, and the algorithm ends.

The purpose of the roll-up building algorithm is to build the upper concept lattice *C*(*K*) on the basis of lower concept lattice $C\left(\bar{K}\right)$, and the drill-down building algorithm is to build the lower concept lattice $C\left(\bar{K}\right)$ on the basis of the upper concept lattice *C*(*K*). From the point of view of granular computing, roll-up building algorithm helps us recognize concepts from fine-grained to coarse-grained, while drill-down building algorithm help us recognize concepts from coarse-grained to fine-grained. Therefore, from the human cognitive thinking process, roll-up and drill-own building algorithms are reversed, and then there is a question to be discussed: whether there is reducibility between these two reverse algorithms.

Now, we consider example 3.

**Example 4 .**

In the layered formal context of example 3, by using the drill-down algorithm, we obtain the lower concept lattice $C\left(\bar{K}\right)$ as shown in Figure 4. Now, we run the roll-up building algorithm on $C\left(\bar{K}\right)$.

**Step 9 s 1 and 2.**

The concepts containing lower attributes in $C\left(\bar{K}\right)$ are arranged in ascending order according to the number of attributes contained in the concepts as follows: $C^{\prime }\left(\bar{K}\right)=\left\{1256,\left(c,c_{1}\right),\left(3,b\left(c,c_{2}\right)\right),\left(4,b\left(c,c_{3}\right)\right),\left(156,a\left(c,c_{1}\right)\right),\left(\left\{\right\},ab\left(c,c_{1}\right)\left(c,c_{2}\right)\left(c,c_{3}\right)\right)\right\}$.

**Step 10 .**

The linear lower subconcepts (1256, (*c*, *c*_1_)), (156, *a*(*c*, *c*_1_)) below node concept (*U*, {}) are rolled up into a new concept (156, *ac*), and the node concept (*U*, {}) is replaced by (*U*, *c*), which are the parent concept (upper bound node) of the new concept (156, *ac*) and the concept (34, *b*) that does not contain lower attributes.

**Step 11 .**

The parallel lower subconcepts (3, *b*(*c*, *c*_2_)), (4, *b*(*c*, *c*_3_)) below node concept (34, *b*) are rolled up into a new concept (34, *bc*). There are no other concepts below (34, *b*) that do not contain lower attributes (*c*, *c*_2_), (*c*, *c*_3_), and then (34, *b*) is updated into (34, *bc*).

**Step 12 .**

The lower attributes (*c*, *c*_1_), (*c*, *c*_2_), (*c*, *c*_3_) in the minimum node concept (empty concept) ({}, *ab*(*c*, *c*_1_)(*c*, *c*_2_)(*c*, *c*_3_)) are integrated, and then we obtain the minimum concept ({}, *abc*).

**Step 13 .**

There is only one layered attribute, and the algorithm ends.

After $C\left(\bar{K}\right)$ is rolled up, the concept lattice is exactly the same as shown in Figure 3. This also shows that the rolling-up and drilling-down algorithms for layered concept lattice have the reducibility.

## 5. Experimentation

The previous example has shown that the roll-up building algorithm and the drill-down building algorithm proposed in this paper are effective. To further show the utility of Algorithms 1 and 2, we implement two algorithms by using MTLAB program language. The computing environment is a PC (Pentium Win7 × 64, Intel (R) 3.4 GHz, RAM 4 GB). The validity verification about the roll-up building algorithm is mainly to investigate the improvement on time consumption by comparing building the concept lattice *C*(*K*) from the lower concept lattice $C\left(\bar{K}\right)$ with building the concept lattice *C*(*K*) directly from the formal context *K*.

**Figure 5:**
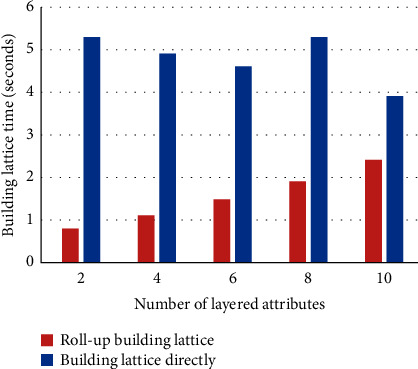
Time comparison between building concept lattice by using roll-up algorithm and building concept lattice directly.

**Figure 6:**
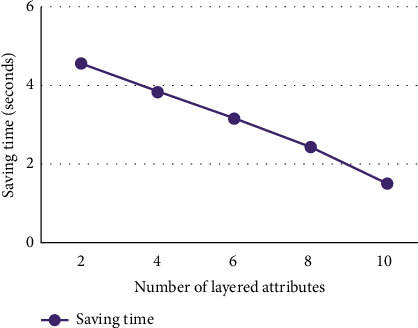
Saving time of roll-up building lattice.

In the experiment, the number of objects is set to 15000, and the total number of attributes is set to 60 (including lower attributes). The number of lower attributes of each layered attribute is set to 5, the number of layered attributes is 2, 4, 6, 8, and 10, respectively, and the total number of lower attributes correspondingly is 10, 20, 30, 40, and 50, respectively, so the number of upper attributes in the lower form context $\bar{K}$ is 52, 44, 36, 28, and 20, respectively. The test data with medium strength filling ratio (|*I*|/(|*G*||*M*|)) 20% are randomly generated. If a layered attribute has no filling value, then its corresponding lower attribute values are all 0. If a layered attribute has filling value, then select randomly a lower attribute to be assigned a value of 1. The improved Bordat algorithm proposed in [12] was adopted for building context lattice directly. The comparison on time consumption building directly the concept lattice from the dataset with building the concept lattice by using roll-up building algorithm is shown in Figure 5, and the time saved by using the roll-up building algorithm to build the concept lattice is shown in Figure 6.

It can be seen from Figures 5 and 6 that using the roll-up building algorithm to build concept lattice *C*(*K*) on the basis of $C\left(\bar{K}\right)$ takes less time than building directly the concept lattice *C*(*K*) from the formal context *K*. When the number of layered attributes is relatively small, the effect of time saving by using roll-up building algorithm is very obvious, and as the number of layered attributes increases, the time saving decreases.

The validity verification about the drill-down building algorithm is mainly to investigate the improvement on time consumption by comparing building the lower concept lattice $C\left(\bar{K}\right)$ from the upper concept lattice *C*(*K*) with building the concept lattice $C\left(\bar{K}\right)$ directly from the formal context $\bar{K}$.

**Figure 7:**
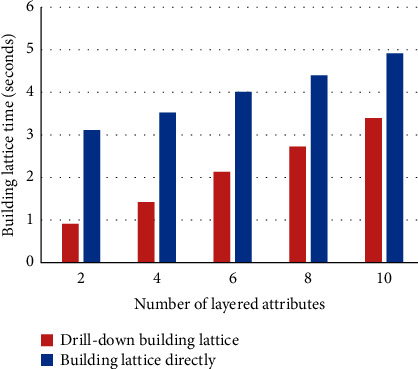
Time comparison between building concept lattice by using drill-down algorithm and building concept lattice directly.

**Figure 8:**
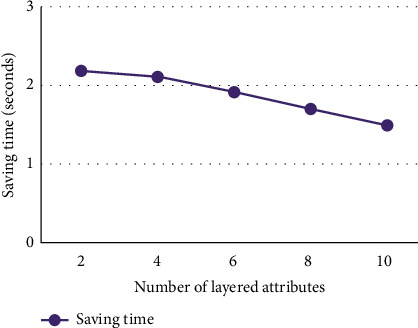
Saving time of drill-down building lattice.

In the experiment, the number of objects is set to 15000, and the total number of attributes is set to 60 (including lower attributes). The number of nonlayered attributes is set to 10, the number of layered attributes is set to 2, 4, 6, 8, and 10, respectively, and the number of lower attributes of each layered attribute is set to 5, so the total number of attributes in the lower formal context $\bar{K}$ is 20, 30, 40, 50, and 60, respectively. The experimental environment and dataset are the same as the roll-up building algorithm experiment. The comparison on time consumption building directly the concept lattice from the dataset with building the concept lattice by using drill-down building algorithm is shown in Figure 7, and the time saved by using the drill-down building algorithm to build the concept lattice is shown in Figure 8.

It can be seen from Figures 7 and 8 that using the drill-down building algorithm to build concept lattice $C\left(\bar{K}\right)$ on the basis of *C*(*K*) takes less time than building directly the concept lattice $C\left(\bar{K}\right)$ from the formal context $\bar{K}$. When the number of layered attributes is relatively small, the effect of time saving by using drill-down building algorithm is very obvious, and as the number of layered attributes increases, the time saving decreases.

## 6. Conclusion

This paper applies the idea of granular computing to build concept lattice in a formal context with complex structure attribute data. When some attributes in a formal context are made up of some subattributes, a layered concept lattice model is established, and the relation between the upper concept lattice based on the original formal context and the lower concept lattice based on the lower formal context is discussed. A roll-up building algorithm that builds the upper concept lattice from the lower concept lattice and a drill-down building algorithm that builds the lower concept lattice from the upper concept lattice are proposed; the time complexity of two algorithms is analyzed. The application of the algorithm and the reducibility of the roll-up building algorithm and the drill-down building algorithm are illustrated by some practical examples. Practical examples and experiments show that the layered concept lattice model can be used to model complex structural data, and the roll-up building algorithm and the drill-down building algorithm proposed in this paper are effective. In future work, we will discuss in detail the reducibility between the roll-up building algorithm and the drill-down building algorithm, the roll-up building algorithm and the drill-down building algorithm for the layered concept lattice in a not-normal layered context. This paper does not discuss the relationship between layered attributes from a quantitative point of view. It also may be another possible research direction to use the analytic hierarchy process to discuss the layered concepts in layered formal context from a quantitative point of view.

## Figures and Tables

**Algorithm 1:**
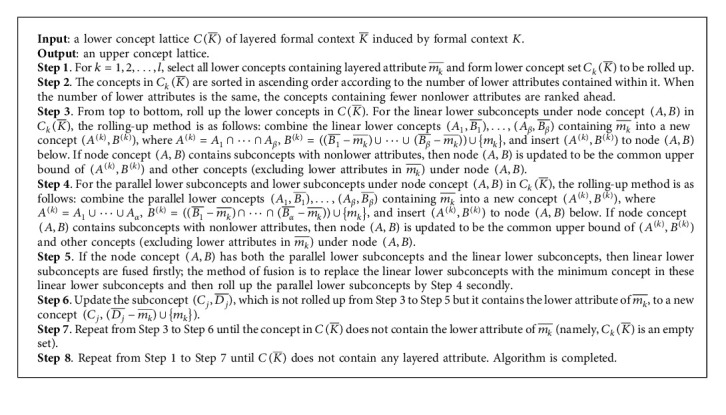
Roll-up building algorithm of layered concept lattice.

**Algorithm 2:**
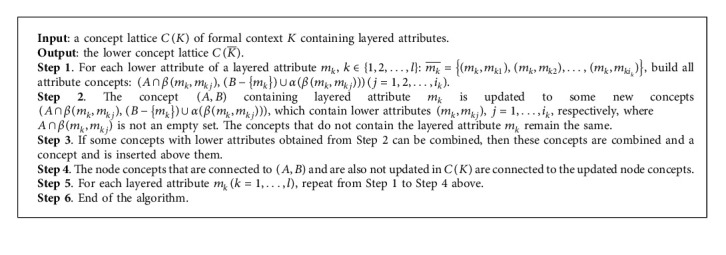
Drill-down algorithm to build the lower concept lattice.

**Table 1:** Layered formal context *K*.

*G*	*a*	*b*	*c*	
			*c*_1_	*c*_2_
1	1	0	0	0
2	0	1	0	0
3	0	0	1	0
4	0	0	0	1
5	1	1	1	0
6	1	1	0	1

**Table 2:** Formal context $\bar{K}$ induced by *K*.

*G*	*a*	*b*	(*c*, *c*_1_)	(*c*, *c*_2_)
1	1	0	0	0
2	0	1	0	0
3	0	0	1	0
4	0	0	0	1
5	1	1	1	0
6	1	1	0	1

**Table 3:** Layered formal context *K*.

*G*	*a*	*b*	*c*		
			*c*_1_	*c*_2_	*c*_3_
1	1	0	1	0	0
2	0	0	1	0	0
3	0	1	0	1	0
4	0	1	0	0	1
5	1	0	1	0	0
6	1	0	1	0	0

**Table 4:** Formal context $\bar{K}$ induced by *K*.

*G*	*a*	*b*	(*c*, *c*_1_)	(*c*, *c*_2_)	(*c*, *c*_3_)
1	1	0	1	0	0
2	0	0	1	0	0
3	0	1	0	1	0
4	0	1	0	0	1
5	1	0	1	0	0
6	1	0	1	0	0

## Data Availability

The data used to support the findings of this study are randomly available according to the method described in the article.
